# Dual-Responsive Alginate Hydrogels for Controlled Release of Therapeutics

**DOI:** 10.3390/molecules24112089

**Published:** 2019-05-31

**Authors:** Xuexia Lin, Qiaoqiao Ma, Jianlong Su, Cui Wang, Ranjith Kumar Kankala, Mingrong Zeng, Honggui Lin, Shu-Feng Zhou

**Affiliations:** 1 Department of Chemical Engineering& Pharmaceutical Engineering, College of Chemical Engineering, Huaqiao University, Xiamen 361021, China; nisingm@gmail.com (Q.M.); sujianlong_123@163.com (J.S.); ranjithkankala@hqu.edu.cn (R.K.K.); mingrong@hqu.edu.cn (M.Z.); 2 Biology department, College of Art and Science, Georgia State University, Atlanta, GA 30303, USA; lorna.cui@hotmail.com; 3 School of Marine Engineering, Jimei University, Xiamen 361021, China

**Keywords:** pH-responsive, thermos-responsive, drug release, antimicrobial efficacy, drug carrier

## Abstract

In this work, with the drug oxytetracycline (OTC) released, cell cytotoxicity and antimicrobial studies of dual-responsive sodium alginate and *N*-Isopropylacrylamide hydrogels (SA/pNIPAAm) with enclosed OTC were investigated. The molecular OTC release was explored with different acid-base conditions and temperature conditions. In order to characterize cell cytotoxicity and antimicrobial efficacy, time-dependent OTC release analysis of different acid-base conditions was performed in SA/pNIPAAm hydrogels. OTC@SA/pNIPAAm hydrogels showed excellent time-dependent antimicrobial efficacy, in which the IC50 values were 50.11 μg mL^−1^, 34.27 μg mL^−1^, and 22.39 μg mL^−1^ among three consecutive days, respectively. Meanwhile, the human cells showed excellent viability at the IC50 dosage of OTC@SA/pNIPAAm (50.11 μg mL^−1^). OTC@SA/pNIPAAm performed in this study indicated that SA/pNIPAAm may serve as drug carriers for sustainable release at a specific concentration and for being employed as substrates for decreasing drug toxicity. Besides, pH-responsive and thermos-responsive SA/pNIPAAm may lead to the better selectivity of drug release in the ideal location or site. Finally, the results demonstrate that the designed, dual-responsive, biocompatible OTC@SA/pNIPAAm hydrogels showed excellent antimicrobial efficacy and may potentially be found to have enormous applicability in the field of pharmaceutics.

## 1. Introduction

Recently, a significant amount of efforts has been made in the search for appropriate drug carriers that could predominantly deliver therapeutic molecules with high efficiency [1,2,3]. Indeed, the ideal drug carrier could offer a suitable way to deliver drugs irrespective of their molecular weights to the target site and hold the control over the drug release predominantly within the physiological environments to fulfill specific therapeutic needs [4]. Hydrogels are considered one of such drug carriers that could be extensively utilized in the fabrication of controlled drug delivery systems, due to their advantageous physicochemical properties and morphological attributes that could mimic the living tissues [5,6]. More often, desired polymeric hydrogels are fabricated by grafting different functionalities that could respond to various external and biological stimuli, such as pH, temperature, electromagnetic radiation, and light, among others [7]. Today, multi-responsive hydrogel systems have [8,9,10], garnered enormous attention in drug delivery, such as their ability to respond to multi-stimuli (for example, pH/ionic strength [11], magnetic/thermal effect, and temperature/electrolyte concentration) [12,13]. Among various stimuli, pH- and temperature-responsiveness have been widely explored [14]. By the application of pH/temperature-responsive polymer, Piaoping Yang group has developed a novel biocompatibility core/shell structured anti-cancer drug for nanotheranostic [15]. Based on *N*-Isopropylacrylamide, N,N-dimethylacrylamide, and sulfamethazineacrylamide, Yuki Hiruta group has developed a pH/temperature-responsive fluorescence polymer probe for cellular uptake [16]. Peng Liu et al. have used thermal-responsive poly (*N*-Isopropylacrylamide) as a gatekeeper”and pH -responsive poly (methacrylic acid) (PMAA) as shells to design a core-shell nanoparticles for targeting and controlling antitumor drug delivery [17]. The application of pH- and temperature hydrogels in drug delivery is required to have acceptable biodegradability and biocompatibility. The degradation of hydrogels is not only important for the removal of empty carriers, but also for the release of active compounds. Hence, there is a continuous demand in developing innovative degradable hydrogels for drug delivery applications.

Sodium alginate (SA), a significant family of polysaccharides, is a polyanionic linear copolymer of 1,4-linked-α-guluronic acid and β-d-mannuronic acid residues, which has been utilized in various biomedical applications, such as principal components of wound healing materials [18], controlled drug delivery systems [19], and constructs for tissue engineering [20,21]. The properties of SA include its soft nature, excellent biocompatibility, biodegradability, enhanced permeation and retention effect, pH-responsive ability and ease of chemical modification, which makes it suitable for the reconstruction of the carrier for drug delivery. However, the applicability of SA in controlled-delivery systems is often limited, as it is prone to enzymatic degradation, thereby lacking some responsive function and persistent stability. To overcome these problems, several attempts have been made by chemically modified SA to graft various desired functionalities. Successful attempts have been reported, involving the grafting copolymerization by synthetic polymers, such as poly (*N*-Isopropylacrylamide) (PNIPAAm) [22], poly (lactic-co-glycolic acid) [23], polyacryl-amide [24], and poly (acrylic acid) [25], with the outcome of the temperature-sensitive and/or pH-sensitive gels.

**Figure 1:**
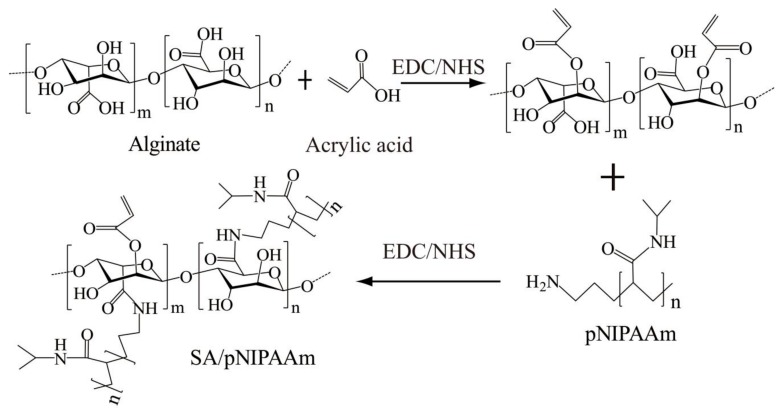
Schematic illustration showing the synthesis of the sodium alginate and *N*-Isopropylacrylamide (SA/pNIPAAm) hydrogel.

Motivated by these facts, SA/pNIPAAm hydrogels were fabricated as drug carriers for the controlled-release of OTC, giving special attention to the promising pH- and thermos-responsive effects (Figure 1). OTC was selected as a model drug since it has been extensively used as an antibiotic and a kind of growth promoter for veterinary drugs. In this work, we have analyzed OTC release, cell cytotoxicity and antimicrobial studies by the application of temperature-sensitive and pH-sensitive SA/pNIPAAm hydrogels as drug carriers. Herein, we focused on the synthetic of pH- and thermos-responsive SA/pNIPAAm hydrogels firstly. pNIPAAm was selected as this thermo-responsive material holds a low critical transition temperature (LCST) of about 32 °C. In addition, due to acrylic acid (AA)’s carboxylic acid groups that can deliver protons at high pH in the surrounding environment, it was selected for adjusting ionic strength and constructing a pH-sensitive sol-to-gel transition [26]. Different techniques, including FTIR spectroscopy, differential scanning calorimetry (DSC), SEM, and NMR, were used to characterize SA/pNIPAAm hydrogels. The pH- and temperature-induced swelling and shrinkage behaviors of hydrogels were also investigated. To prove the suitability of SA/pNIPAAm hydrogel for drug delivery system, the degradation of SA/pNIPAAm hydrogels and the drug entrapment efficiency (EP%) were evaluated. Since OTC has been extensively used as an antibiotic and growth promoter [27], it was loaded as the model drug in this study. For the drug release study, the release kinetics of OTC were performed at 37 °C with pH values ranging from 1.91 to 11.65. Finally, the cytotoxicity, as well as antimicrobial properties, were investigated. The results pointed out that SA/pNIPAAm hydrogels may be employed as drug carrier for prolonging drug release, and OTC@SA/pNIPAAm have low cytotoxicity and high antimicrobial ability.

## 2. Materials and Methods

### 2.1. Reagents and Materials

SA (viscosity (Pas): 10 g/L, 20 °C ≥ 0.02) was obtained from Xilong Scientific Co., Ltd. (Shantou, China). OTC was purchased from Solarbio Life Sciences Co., Ltd. (Beijing, China). pNIPAAm was procured from Shanghai ZZBIO CO. Ltd. (Shanghai, China). EDC and calcium chloride anhydrous were purchased from Shanghai Macklin Biochemical Co., Ltd (Shanghai, China). GA (25% *w/w*) solution, NHS was purchased from Aladdin Reagent Co., Ltd (Shanghai, China).

0.01 M phosphate buffer saline (PBS) (pH = 7.4) was prepared by 0.01 M dibasic sodium phosphate and 1.7 mM potassium phosphate monobasic. Adding HCl or NaOH in 0.01M PBS solution to adjust different pH values.

### 2.2. Preparation of SA/pNIPAAm and OTC@SA/pNIPAAm

SA/pNIPAAm was prepared by using EDC/NHS as initiators. In brief, SA solution (3%, 500 mL) was prepared by adding deionized water, followed by adding 1 mL of acrylic acid and 80 mg of EDC. The mixture was stirred at room temperature under 500 rpm. After 2 h, the solution was added with 40 mg of NHS and 500 mg of pNIPAAm and continuously stirred at room temperature at 500 rpm for 24 h. Finally, the solution was dialyzed for 5 days to obtain pure SA/pNIPAAm. After that, SA/pNIPAAm was evaporated with gentle heating and continuous stirred until to obtain 3% SA/pNIPAAm. OTC (500 μg/mL) was added to the prepared 3% SA/pNIPAAm solution. The solution was stirred until the drug was evenly dispersed and then dialyzed for 2 days to obtain pure OTC@SA/pNIPAAm hydrogels. Subsequently, CaCl_2_ (300 mM) was used to form hydrogels. Then, the OTC@SA/pNIPAAm hydrogels were freeze-dried for 36 h before next experiment.

### 2.3. Physical Characterization

The chemical functionalities of the dry samples were recorded using the Fourier transform infrared (FT-IR) spectrometer (Thermo Scientific, Nicolet, Waltham, MA, USA). The samples were prepared by sublimation of hydrogels using liquid nitrogen and freeze-dried under vacuum for 36 h. Further, the dried samples were ground into fine powder and subjected to analysis by the KBr pellet method. The glass-transition temperatures (Tg) of dried samples were measured on a Netzsch DSC (Netzsch-Gerätebau GmbH, Selb, Germany) at a scan rate of 3 °C/min under a dry nitrogen atmosphere. The weight of the sample was within the range of 6–10 mg, while the temperature was within the range of 20~150 °C.

To observe the morphologies of the surface of different pH values and temperature ranging from 28 to 37 °C of the SA/pNIPAAm hydrogels, the wet SA/pNIPAAm hydrogels were frozen directly in cold stage (−80 °C) and freeze-dried, then SA/pNIPAAm hydrogels were sputtered with gold for 60 s before observation on a JSM-5600LV emission scanning electron microscopy (SEM, JEOL, Tokyo, Japan).

### 2.4. Characterization of SA/NIPAAm by NMR

Solid-state NMR experiments of SA/pNIPAAm were carried out using a Bruker AVANCE III 400 spectrometer (Bruker, Faellanden, Switzerland) with a frequency of 100.38 MHz (magnetic field strength 9.4 T) for ^13^C. The experiments were carried out using a 4 mm MAS (magic angle spinning) probe at a spinning speed of 5 kHz at room temperature (298 K). ^13^C CPTOSS NMR (cross-polarization experiment with Total Suppression of Sidebands) (Bruker, Faellanden, Switzerland) spectra were recorded using a contact time of 3 ms. The recycle delay for SA/pNIPAAm was 2 s.

### 2.5. Swelling-Deswelling and Degradation Behaviors of SA/pNIPAAm Hydrogels

To assess the pH- and temperature-dependent swelling behavior of hydrogels, the swelling behavior of SA/pNIPAAm hydrogel was investigated in different buffers (pH = 1.99~11.65) (*I* = 0.1 M) maintained at different temperature (28~37 °C). At an appropriate interval, excess water on the surfaces of SA/pNIPAAm hydrogel were removed by wet filter paper, and the weight of the swollen samples was measured. The swelling ratio (SR) was calculated using the following expression (Equation (1)):

(1) $$SR=\frac{W_{t}-W_{0}}{W_{0}}$$

where *W_t_* and *W_0_* are the weights of the swollen and initial samples, respectively.

The SA/pNIPAAm hydrogels were pre-equilibrated swollen at 28 °C and then placed into 37 °C baking oven. Further, the weight of the samples was measured at an appropriate interval. The deswelling ratio (DR%) was calculated as the following (Equation (2)):

(2) $$DR\%=\frac{W_{0}-W_{t}}{W_{0}}\times 100$$

where *W_t_* and *W_0_* are the same as described above.

For degradation analysis, the pre-equilibrated swollen SA/pNIPAAm hydrogels were immersed into the respective medium during 156 h at 37 °C. At an appropriate interval, excess water on the surface of hydrogels was removed, and the hydrogels were dried and weighed. The degradation ratio (DGR%) was calculated by the following (Equation (3)):

(3) $$DGR=\frac{W_{0}-W_{e}}{W_{0}}\times 100\%$$

where *W_e_* and *W_0_* are the weights of initial and immerged hydrogels, respectively.

### 2.6. Entrapment Efficiency In Vitro

The EP% of OTC was determined according to the following method. 500 μg/mL of OTC was added to the prepared 3% SA/pNIPAAm solution (pH = 7.4) at 28 °C. The solution was stirred until the drug was evenly dispersed and dialyzed for 2 days to obtain pure OTC@SA/pNIPAAm. CaCl_2_ (300 mM) was used to form hydrogels. The SA/pNIPAAm contained OTC was freeze-dried for 36 h. The freeze-dried samples were ground evenly and then dissolved in PBS buffer (100 mM) to the final concentrations of OTC@SA/pNIPAAm at 300 μg/mL. The 50 μg/mL OTC@SA/pNIPAAm solution was continuous shaking 30 min. The aqueous solution was filtered and analyzed using a UV spectrophotometer at the fixed max value of 269 nm. The results of % encapsulation efficiency were calculated as (Equation (4)):

(4) $$EP\%=\frac{W_{OTC}}{W_{}}\times 100$$

where *W_OTC_* and *W* are the weight of experimental OTC loading and the weight of theoretical OTC loading, respectively.

### 2.7. OTC Release In Vitro

OTC@SA/pNIPAAm (2 mL, 250 μg/mL) was added to PBS buffer at different pH values and different temperature conditions. At regular time intervals, the OTC@SA/pNIPAAm hyrdrogels were weighted, and the aliquots of OTC release samples were eluted from OTC@SA/pNIPAAm hyrdrogels by centrifugal. The water on the OTC@SA/pNIPAAm hyrdrogels surface was dried with absorbent paper. Then, the remaining OTC@SA/pNIPAAm hyrdrogels was replenished to the pre-warmed fresh PBS buffer with the different values and under different temperature. Meanwhile, 300 μL of eluted solution was measured by UV-Vis spectra at 269 nm at each time interval.

### 2.8. Cytotoxicity

Cytotoxic effect of the OTC@SA/pNIPAAm was studied on the most commonly used human umbilical vein endothelial cells (HUVECs) using the cell counting kit-8 kit (CCK-8, Dojindo Laboratories, Kumamoto, Japan). A stock solution of OTC@SA/pNIPAAm (1.0 mg/mL) was serially diluted by a medium into the desired concentrations (0, 25, 50, 100, 200, 250, 500 μg/mL). After 24 h of culture at 37 °C, OTC@SA/pNIPAAm hydrogels (300 μg/mL) were added into the fresh medium, and the mixture were replaced with the media in the wells. After 72 h of incubation, the viability of cells in different treatment conditions was enumerated using the CCK-8 for every 24 h using the Multi-function microplate reader.

### 2.9. Antimicrobial Studies

To evaluate the antimicrobial activity of the OTC@SA/pNIPAAm hydrogels, gram-positive bacteria species *E. coli* BL21 were used as a model. *E. coli* BL21 were grown overnight in the Luria-Bertani (LB) liquid medium at 37 °C and harvested at the exponential growth phase via centrifugation at 3000 rpm for 5 min, and then discarded the supernatant. Then, the bacteria were resuspended in fresh medium. The bacterial concentration was determined by measuring the optical density (OD) at a wavelength of 600 nm. Further, the diluted bacteria solution (200 μL, 1 × 10^5^ CFU mL^−1^) was added with the OTC@SA/pNIPAAm to reach a final concentration of 50 μg/mL into 96-well plate, incubated at 37 °C. Three sets of the same solution as described above are prepared in parallel. Three controls were performed in parallel by the medium with OTC, SA, and the only medium, respectively. Finally, the concentrations of bacteria solution were tested by measuring the OD at a wavelength of 600 nm at regular time. The antibacterial activities of OTC@SA/pNIPAAm were evaluated by Ultraviolet-Visible (UV) Spectrophotometer (Shimadzu Corp., Kyoto, Japan) at OD 600 nm.

## 3. Results and Discussion

### 3.1. Physical Characterization

**Figure 2:**
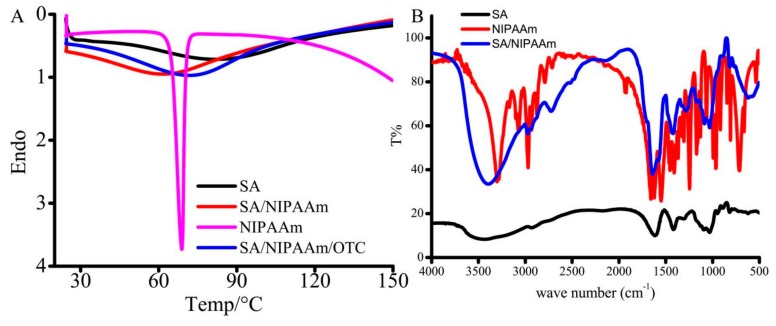
(**A**) Differential scanning calorimetry (DSC) of SA, NIPAAm, SA/NIPAAm, and SA/NIPAAm/OTC. (**B**) Fourier transform infrared (FT-IR) spectra of SA, NIPAAm, and SA/NIPAAm.

Initially, SA/pNIPAAm hydrogels were prepared by EDC/NHS-based polymerization approach. Furthermore, the thermal behaviors of the SA, pNIPAAm, SA/pNIPAAm, and SA/pNIPAAm/OTC were recorded using DSC to explore the thermos-responsive ability of the polymeric construct (Figure 2A). The glass-transition temperature (Tg) values of SA, NIPAAm, SA/pNIPAAm, and SA/pNIPAAm/OTC were 82.6, 68.79, 60.97, and 70.76 °C, respectively, indicating that the gel-to-sol phase transition occurred with a decline of temperature. Moreover, it was evident from the results that the Tg value of SA/pNIPAAm was reduced compared to the individual polymers SA and NIPAAM, demonstrating the deprived thermal stability of SA/pNIPAAm composite hydrogel due to the grafting of pNIPAAm into SA backbone. However, the Tg value of SA/pNIPAAm/OTC increased to 70.76 °C, which was higher than pure SA/pNIPAAm, indicating the successful encapsulation of guest molecules OTC in the frameworks of SA/pNIPAAm hydrogels.

Further, the chemical functionalities were illustrated by FT-IR spectra of SA, SA/pNIPAAm, and pNIPAAm (Figure 2B). The strong and broad band at 3416 cm^−1^, could be attributed to N-H stretching vibration of pNIPAAm in SA/pNIPAAm hydrogels. In addition, it has shown other corresponding peaks, which could be ascribed to amide I band (1646 cm^−1^) assigning to C=O stretching of pNIPAAm, and amide II band (1576 cm^−1^) to N-H vibration. A little band at around 1711 cm^−1^ could be ascribed to carboxyl stretching vibration of SA in SA/pNIPAAm. These results demonstrate that the appropriate chemical functional groups of SA and pNIPAAm were present in the SA/pNIPAAm composite hydrogel.

**Figure 3:**
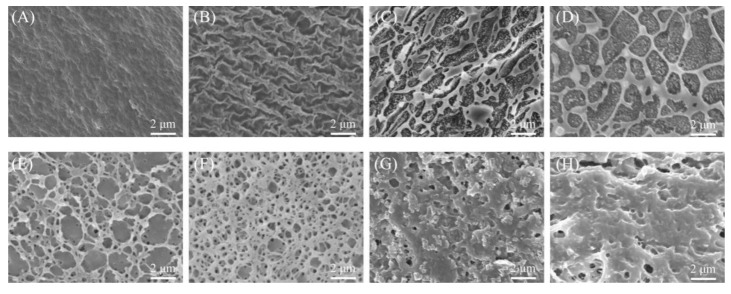
SEM photograph of SA/pNIPAAm hydrogel at varied pH values of (**A**) 1.91, (**B**) 6.03, (**C**) 7.40, and (**D**) 10.96 (constant temperature of 30 °C), and temperatures of (**E**) 28 °C, (**F**) 32 °C, (**G**) 35 °C, and (**H**) 37 °C (constant pH of 7.40).

Figure 3 shows the morphological attributes of SEM micrographs of SA/pNIPAAm at different pH values and temperature ranging from 28 to 37 °C. Obviously, the SA/pNIPAAm hydrogels at high pH values have shown that the highly porous with interconnected windows throughout. At the low pH value, the porous architectures were highly dense with small-sized pores. In hydrogel networks, the crosslinking density is of importance because of its effect on the mechanical properties of these materials, such as swelling, deswelling, and degradation. With different pH conditions, there are different swollen polymer volume fraction and Flory polymer-solvent interaction parameters [28,29]. A small change of the Flory polymer-solvent interaction parameters or/and swollen polymer volume fraction will result in large changes of the crosslinking density in Figure 3. The alternative crosslinking density results in the variation of the stress, tensile strength and the elongation. Therefore, there are different the morphology of SA/pNIPAAm hydrogels were observed. These results support the FT-IR and DSC analysis, and it also can demonstrate that an expanded network could be generated by electrostatic repulsions of carboxyl and amide functionalities of SA/pNIPAAm. In addition, it was found that the mesh size of the SA/pNIPAAm hydrogels decreased with increasing the temperature from 28 °C to 37 °C. It was notable that the mesh size declined sharply and the structure of SA/pNIPAAm hydrogels were compacted between 33 °C and 35 °C. The variations of temperature would have resulted from different crosslinker contents and the Flory polymer-solvent interaction parameters. And it suggested the volume phase transition temperature of hydrogels was from 28 °C to 35 °C. It can be predicted that the water molecules or small molecules could be conveniently diffused out due to such small pores. The results also indicated that the temperature have important effect on the swelling behaviors of SA/pNIPAAm hydrogels. All of the above demonstrated that SA/pNIPAAm hydrogels have the obvious pH and temperature sensitivity.

**Figure 4:**
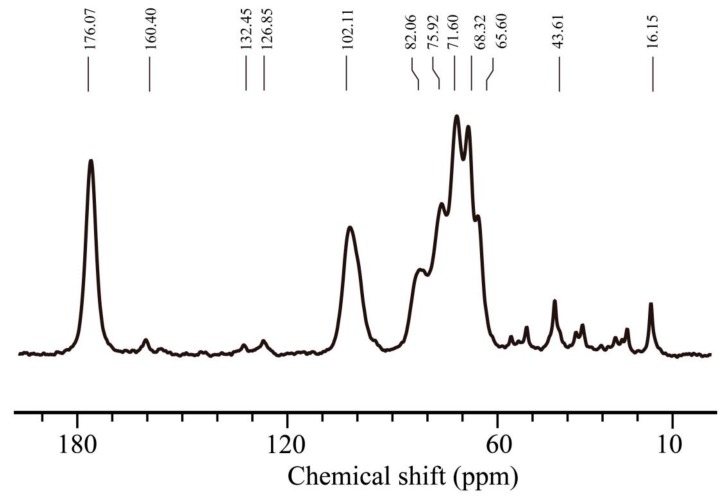
^13^C-NMR spectrum of SA/pNIPAA was obtained by solid-state NMR.

Figure 4 is the solid-state NMR spectrum of SA/pNIPAAm hydrogels. The resonance centered at 176.07 ppm is corresponded to the ester carbonyl carbon (–C=O). At 160.40 ppm the resonance is due to amide carbon (–CON–). At 132.45 ppm and 126.85 ppm the resonance is due to alkene carbon (–C=C–). At 102.11 ppm the resonance is due to methine carbon attached to the carbonyl group. In the range of 70–80 ppm, resonances of methine carbon of alginate and pNIPAAm are clearly observed. The methylene carbons of pNIPAAm resonated in the range of 20–40 ppm.

### 3.2. pH- and Thermos-Responsive Swelling Behavior

**Figure 5:**
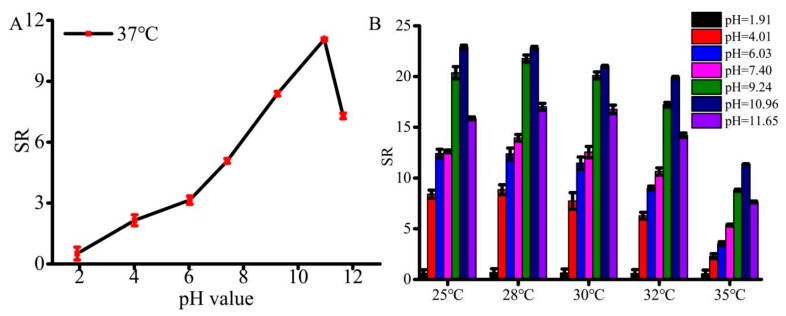
(**A**) Maximum SR of SA/NIPAAm hydrogels in phosphate-buffered saline (PBS) buffer with different pH values under 37 ℃. (**B**) SR of SA/NIPAAm hydrogels for the responsiveness of temperature and pH values.

The equilibrium of hydrogel swelling significantly influences the encapsulation as well as the release of guest molecules from the hydrogel frameworks. Therefore, the swelling behaviors of SA/pNIPAAm hydrogel in response to the environmental factors, such as pH and temperature, were investigated. The SA/NIPAAm hydrogels are composed of crosslinked anionic SA, AA and pNIPAAm. As is well known, most of polyanionic hydrogels are sensitive to pH of solutions. Figure 5A shows variation of the maximum SR (They are calculated according to Equation (1)) of the SA/NIPAAm hydrogels with different pH values. It can be found that the maximum SR of SA/NIPAAm hydrogels is less than 0.6 folds at pH ≥ 2 under 37 °C. The maximum SR increases evidently to 12 folds with increasing pH from 6.03 to 10.96 and then rapidly decreases with further increasing pH to 11.65. We deduced that both AA and SA of –COO– group are responsible for swelling of SA/NIPAAm hydrogels. While most of the –COO– groups can be protonated in acidic solution, leading to the low SR. The –COO– groups are gradually ionized and the electrostatic repulsion increases with increasing pH. Hence, the maximum SR raises. Therefore, the SA/NIPAAm hydrogels are pH-sensitive. As shown in Figure 5B, the highest SR was observed at 25 °C and 28 °C, while the lowest of SR was at 37 °C. The highest SR can reach up to 22-fold at 28 °C and pH=10.96. The swelling ratios was little changed when the temperature 25 °C and 28 °C, and then swelling ratios gradually decreased with increasing temperature. These results indicated that the SA/pNIPAAm composite hydrogel had retained its temperature sensitivity. Furthermore, the SR decreasing rate of SA/pNIPAAm was slower in the case of temperature above 32 °C than that of below 32 °C. The possible reason might be that the LSCT of pNIPAAm is about 32 °C. In addition, the stronger the hydrogen bond at high temperature between –COOH in the SA and –CONH– in the pNIPAAm, the higher polymer-polymer interactions leading to compact structures. Further, these results were confirmed by the SEM observations. Figure 5B also showed that at the same temperature range, swelling ratios of SA/pNIPAAm hydrogels at alkaline condition were higher than that at the acidic conditions. Moreover, the pH value of 1.91 has resulted in the lowest swelling ratio, while the highest maximum swelling ratio was shown at pH = 10.96. The reason might be the ionization property of SA/pNIPAAm hydrogels and the substitution of sodium ions for protons improves the water solubility of SA/pNIPAAm. The similar tendency was found at other temperatures in the same conditions. Interestingly, these composite hydrogels resulted in pH-responsive at a high temperature than that at a low temperature. In addition, to investigate the effect of the environmental pH value, the morphologies of SA/pNIPAAm hydrogels at the pH values of 1.91, 6.03, 7.40, and 10.96, were investigated by SEM. Figure 3 shows that the pore sizes decreased and tended to form sheet structure with decreasing the pH value, except at pH = 7.40. At the pH = 1.91, the morphology of SA/pNIPAAm hydrogels was the compact structures, resulting in the low swelling.

### 3.3. Swelling-Deswelling Kinetics of Hydrogels

**Figure 6:**
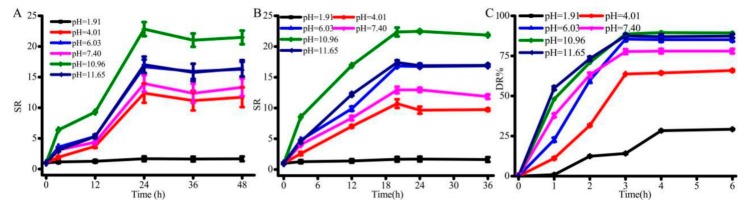
Swelling kinetics of SA/NIPAAm hydrogel at (**A**) 28 °C and (**B**) 37 °C, and (**C**) deswelling kinetics of SA/NIPAAm hydrogel at 37 °C.

To investigate how fast that SA/pNIPAAm hydrogel responds to the external stimuli, the swelling-deswelling kinetics were investigated. Figure 6A,B show the changes in swelling ratio at 28 °C and 37 °C, ranged from pH = 1.91 to pH = 11.65. It was observed from the results that SR of SA/pNIPAAm reached an equilibrium swelling status within 24 h at 28 °C, and 20 h at 37 °C. The gradually reached equilibrium swelling status was possibly due to the polymer-polymer interactions caused by –COOH in the SA and –CONH– in the pNIPAAm reduced swelling rate. In comparison, the temperature at 37 °C over 28 °C could enhance the hydration of SA/pNIPAAm hydrogels, resulting in the raised swelling rate although the architectures of SA/pNIPAAm hydrogels were compact. The difference in the swelling ratio was also found at different pH values. A lower pH value could result in a slower equilibrium swelling status. Moreover, the morphology of SA/pNIPAAm hydrogel fabricated at a lower pH has shown smaller net pore sizes and tended to form sheet-like architectures, which resulted in the decrease of the overall swelling rate of the composites (Figure 3A–D and Figure 5). The variations of swelling behaviors are the result of alternative crosslinking density which bring the different of the stress, tensile strength and then the elongation redistributed to different amount of reinforcement points [28,30]. Figure 6C shows that the deswelling kinetics of SA/pNIPAAm hydrogels at 37 °C exhibited the equilibrium deswelling state at about 3 h. In a range of pH value from 1.91 to 11.65, the highest deswelling was at pH = 11.65 and pH = 10.96. The deswelling rates of SA/pNIPAAm hydrogels gradually reduced with gradually decreasing the pH value. These consequences could also be confirmed by SEM observations. Similarly, the SA/pNIPAAm hydrogels fabricated at the low pH value have shown low DR% due to their dense structure.

### 3.4. Evaluation of EP%, Degradation Efficiency and OTC Drug Release In Vitro

**Figure 7:**
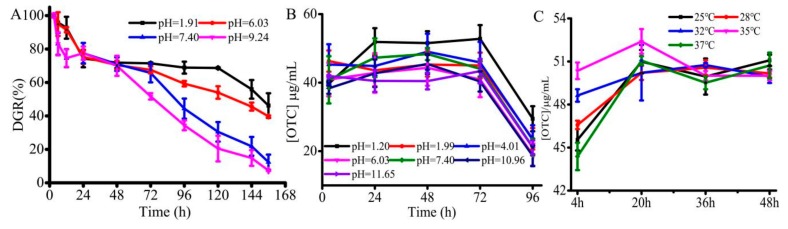
(**A**) The time-dependent degradation of SA/pNIPAAm under 37 °C. (**B**) The time-dependent controlled OTC from OTC@SA/pNIPAAm under 37 °C. (**C**) The time-dependent controlled OTC from OTC@SA/pNIPAAm under pH = 7.40.

Further, various characterization including the EP% and degradation efficiency were explored as these parameters play a crucial role in drug delivery application. According to equations 3, the EP% of OTC was around 80.5% at 28 °C and pH = 7.4, demonstrating that the designed hydrogels at appropriate synthetic conditions have shown high encapsulation efficiency, which could significantly decrease the drug wastage. In addition to encapsulation and release of guest molecules, the degradation of designed formulation plays a significant role in fabricating functional delivery systems. Therefore, the degradation of freeze-dried SA/pNIPAAm hydrogel at pH = 1.20 and 7.40 at 37 °C was evaluated. The rationale behind the selection of the buffer systems at such pH values is that the respective pH values were similar to the pH value of the stomach and intestine. As shown in Figure 7A, the weights of SA/pNIPAAm hydrogels were gradually degraded at pH = 1.20 during 156 h, and the hydrogel DGR was achieved 0.5591. DGR is calculated according to equations 2. SA/pNIPAAm hydrogel was slightly degraded at pH = 7.40. The degradation rates were slightly augmented with time. It could be deduced that the long-time degradation of SA/pNIPAAm hydrogel was due to the change in the overall architecture of hydrogel by acrylic acid and increased the acid resistance of the hydrogel. The results displayed that the synthetic SA/pNIPAAm would be used as a drug delivery carrier and for drug sustained-release. Because of the pre-warmed fresh PBS was replaced the OTC@SA/pNIPAAm hydrogel elution solution, the Y-axis in Figure 7B is represented by the release concentrations of OTC from OTC@SA/pNIPAAm hydrogel every 24 h. The results exhibited an initial slow release of OTC in the pH range from 1.2 to 11.65 during the initial 24 h, and then the release was maintained in a range of 40–45 μg/mL during 72 h. After 72 h, the increased OTC release may be responsible the augmented SA/pNIPAAm hydrogel degradation. The highest release rate at pH = 1.20 might be resulted from the rapid SA/pNIPAAm hydrogel degradation, while the drug release is predominantly due to swelling at other pH values. As shown in Figure 7C, it can be found that the levels for OTC release were increased with raising the temperature ranging from 25 °C to 35 °C during 4 h, indicating that OTC@SA/pNIPAAm hydrogels have thermos-responsive. The shortest equilibrium time of OTC release was at 32 °C and 35 °C and the OTC release concentration maintained about 50 µg/mL. Moreover, the time for OTC release equilibrium is about 20 h regardless of the effect of temperature. Indeed, the delivery of drugs from any formulation is a gradual process that happens to be favorable due to various factors, such as diffusion at the on-site of infection. OTC@SA/pNIPAAm hydrogels offers an advantage of releasing OTC at the slower rate, and therefore giving longer time to maintain a certain concentration of OTC in the system. These results indicated that the synthetic SA/pNIPAAm hydrogel would be used as a delivery carrier for sustained release of drugs.

### 3.5. Cytotoxicity Studies and Antimicrobial Studies

**Figure 8:**
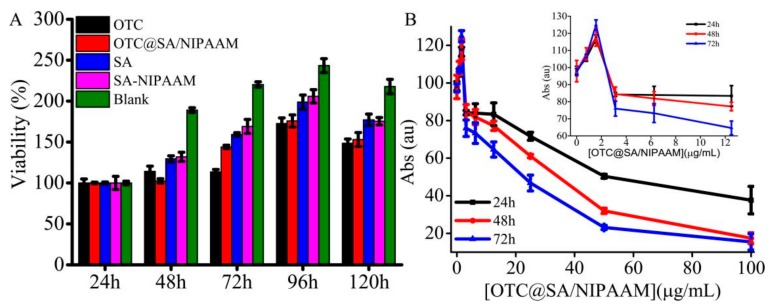
(**A**) Cytotoxicity of SA/pNIPAAm at 37 °C. (**B**) The dependence of absorbance of gram-positive bacteria on the concentration of OTC@SA/pNIPAAm.

To further demonstrate the biocompatibility of designed composites, the samples, OTC, OTC@SA/pNIPAAm, SA/pNIPAAM, and SA were exposed to HUVEC cells and compared the viability of cells with the negative control (media alone). The cell cultured with medium only was set as the control. Figure 8A shows that the samples have no significant influence on the viability of cells cultured during 120 h in the control as well as OTC (40 μg mL^−1^) and OTC@SA/pNIPAAm (50 μg mL^−1^) samples. Notably, the cells cultured with 50 μg mL^−1^ OTC@SA/pNIPAAm, SA, and SA/pNIPAAm, have shown the highest viable rates around 175, 198, and 205%, respectively. The viability curve of cells by SA/pNIPAAM, SA and medium only were similar, and the viability decreased a little when cells were cultured by SA/pNIPAAM or SA. The higher viability of cells cultured by SA/NIPAAM or SA also illustrated that the biocompatibility of SA/pNIPAAm hydrogel was comparable to or even higher than that of SA, indicating that the designed formulation can be suitable as a drug carrier.

Further, the antimicrobial efficacy of OTC from OTC@SA/pNIPAAm was explored. In order to demonstrate the appropriate amount of the OTC@SA/pNIPAAm for antimicrobial activity study, the bactericidal properties of OTC@SA/pNIPAAm were initially evaluated (Figure 8B). It was observed from the results that the increase of OTC@SA/pNIPAAm concentration within 0~100 μg mL^−1^ resulting in decreasing viability of gram-positive bacteria, demonstrating that the release of OTC as well as its antimicrobial efficacy were dose-dependent. The bacteria were increased when the concentration of OTC@SA/pNIPAAm was 1.00 μg mL^−1^. The reasons would be that the bacteria themselves are still reproductive under insufficient drug dosage. Moreover, an OTC@SA/pNIPAAm concentration of 50.11 μg mL^−1^ was effective in gram-positive bacteria on the first day, with a viable rate of 50%. While the concentrations of OTC@SA/pNIPAAm were 34.27 μg mL^−1^ on the second day and 22.39 μg mL^−1^ on the third day. The concentration of OTC@SA/pNIPAAm reduced with the increase of treatment time, indicating that the designed formulation has shown high antimicrobial activity, which could be due to the sustained release OTC from OTC@SA/pNIPAAm. Furthermore, using a lower concentration of OTC@SA/pNIPAAm would be beneficial to the metabolism and degradation of drugs, and could be more conducive to human health.

## 4. Conclusions

In this study, the SA/pNIPAAm hydrogels prolonging drug release has been explored at 37 °C with the pH ranging from 1.91 to 11.65. Firstly, SA/pNIPAAm hydrogels were synthesized. The synthetic SA/pNIPAAm hydrogels have exhibited pH and temperature sensitivity. SA/pNIPAAm hydrogels have shown excellent degradation, swelling, and deswelling abilities, indicating the potential of the synthetic hydrogels as an ideal drug carrier. Secondly, we have explored the drug release of the applied SA/pNIPAAm. The high biocompatible, antimicrobial ability of OTC@SA/pNIPAAms were found to be similar in comparison with OTC, while the cytotoxicity o**f** OTC@SA/pNIPAAms were greatly reduced. Important differences were found in drug release, due to the degradation, swelling and deswelling properties of SA/pNIPAAm. Thus, the long-term sustained drug release at a specific concentration was found. Furthermore, these biocompatible OTC@SA/pNIPAAm constructs have shown excellent antimicrobial efficacy towards the gram-positive bacteria. Together, we believe that this biocompatible, sustained release formulations, which based on hydrogel composites will find a potential way in the field of pharmaceutics.

## References

[1] Pundir S., Badola A., Sharma D. (2013). Sustained release matrix technology and recent advance in matrix drug delivery system: A review. Int. J. Drug Res. Tech..

[2] Hu C., Chen Z., Wu S., Han Y., Wang H., Sun H., Kong D., Leng X., Wang C., Zhang L. (2017). Micelle or polymersome formation by PCL-PEG-PCL copolymers as drug delivery systems. Chinese Chem. Lett..

[3] Liu C.-G., Han Y.-H., Zhang J.-T., Kankala R.K., Wang S.-B., Chen A.-Z. (2019). Rerouting engineered metal-dependent shapes of mesoporous silica nanocontainers to biodegradable Janus-type (sphero-ellipsoid) nanoreactors for chemodynamic therapy. Chem. Eng. J..

[4] Ramasamy T., Ruttala H.B., Gupta B., Poudel B.K., Choi H.-G., Yong C.S., Kim J.O. (2017). Smart chemistry-based nanosized drug delivery systems for systemic applications: A comprehensive review. J. Control. Release.

[5] Li Y., Wang X., Wei Y., Tao L. (2017). Chitosan-based self-healing hydrogel for bioapplications. Chinese Chem. Lett..

[6] Li J., Mooney D.J. (2016). Designing hydrogels for controlled drug delivery. Nat. Rev. Mater..

[7] Liu Q.H., Wang G., Li M., Liu J., Ding X., Huang W., Gao W., Wu H. (2018). A photocleavable low molecular weight hydrogel for light-triggered drug delivery. Chinese Chem. Lett..

[8] Kankala R.K., Wang S.-B., Chen A.-Z., Zhang Y.S., Conde J. (2018). Chapter 2 - Self-Assembled Nanogels: From Particles to Scaffolds and Membranes. Handbook of Nanomaterials for Cancer Theranostics, 1.

[9] Ghaffari R., Eslahi N., Tamjid E., Simchi A. (2018). Dual-sensitive hydrogel nanoparticles based on conjugated thermoresponsive copolymers and protein filaments for triggerable drug delivery. Acs Appl. Mater. Interfaces.

[10] Narendra S., Lee K., Sung D. (2014). In situ gelling pH- and temperature-sensitive biodegradable block copolymer hydrogels for drug delivery. J. Control. Release.

[11] Shang J., Theato P. (2018). Smart composite hydrogel with pH-, ionic strength- and temperature-induced actuation. Soft Matter.

[12] Didehban K.H., Mohammadi L., Azimvand J. (2017). Preparation of RGO/Fe_3_O_4_/poly (acrylic acid) hydrogel nanocomposites with improved magnetic, thermal and electrochemical properties. Mater. Chem. Phys..

[13] Chen Y., Wang Y., Shi X., Jin M., Cheng W., Ren L., Wang Y. (2017). Hierarchical and reversible assembly of graphene oxide/polyvinyl alcohol hybrid stabilized Pickering emulsions and their templating for macroporous composite hydrogels. Carbon.

[14] Rasib S.Z.M., Ahmad Z., Khan A., Akil H.M., Othman M.B.H., Hamid Z.A.A., Ullah F. (2018). Synthesis and evaluation on pH- and temperature-responsive chitosan-p(MAA-co-NIPAM) hydrogels. Int. J. Biol. Macromol..

[15] Lv R., Yang P., He F., Gai S., Yang G., Dai Y. (2015). An imaging-guided platform for synergistic photodynamic/photothermal/chemo-therapy with pH/temperature-responsive drug release. Biomaterials.

[16] Hiruta Y., Funatsu T., Matsuura M., Wang J., Ayano E., Kanazawa H. (2015). pH/temperature-responsive fluorescence polymer probe with pH-controlled cellular uptake. Sensor Actuat. B Chem..

[17] Zeng J., Du P., Liu L., Li J., Tian K., Jia X. (2015). Superparamagnetic Reduction/pH/Temperature Multistimuli-Responsive Nanoparticles for Targeted and Controlled Antitumor Drug Delivery. Mol. Pharm..

[18] Raguvaran R., Manuja B.K., Chopra M., Thakur R., Anand T., Kalia A., Manuja A. (2017). Sodium alginate and gum acacia hydrogels of ZnO nanoparticles show wound healing effect on fibroblast cells. Int. J. Biol. Macromol..

[19] Treenate P., Monvisade P. (2017). In vitro drug release profiles of pH-sensitive hydroxyethylacryl chitosan / sodium alginate hydrogels using paracetamol as a soluble model drug. Int. J. Biol. Macromol..

[20] Marrella A., Lagazzo A., Barberis F., Catelani T., Quarto R., Scaglione S. (2017). Enhanced mechanical performances and bioactivity of cell laden-graphene oxide/alginate hydrogels open new scenario for articular tissue engineering applications. Carbon.

[21] Thakur S., Sharma B., Verma A., Chaudhary J., Tamulevicius S., Thakur V.K. (2018). Recent progress in sodium alginate based sustainable hydrogels for environmental applications. J. Clean. Prod..

[22] Liu M., Song X., Wen Y., Zhu J.-L., Li J. (2017). Injectable thermoresponsive hydrogel formed by alginate-*g*-poly(*N*-isopropylacrylamide) that releases doxorubicin-encapsulated micelles as a smart Drug Delivery System. Acs Appl. Mater. Interfaces.

[23] Chai F., Sun L., He X., Li J., Liu Y., Xiong F., Ge L., Webster T.J., Zheng C. (2017). Doxorubicin-loaded poly (lactic-co-glycolic acid) nanoparticles coated with chitosan/alginate by layer by layer technology for antitumor applications. Int. J. Nanomed..

[24] Zhou Q., Kang H., Bielec M., Wu X., Cheng Q., Wei W., Dai H. (2018). Influence of different divalent ions cross-linking sodium alginate-polyacrylamide hydrogels on antibacterial properties and wound healing. Carbohyd. Polym..

[25] Pakdel P.M., Peighambardoust S.J. (2018). A review on acrylic based hydrogels and their applications in wastewater treatment. J. Environ. Manage..

[26] Yin M.-J., Yao M., Gao S., Zhang A.P., Tam H.-Y., Wai P.K. (2016). Rapid 3D Patterning of Poly (acrylic acid) Ionic Hydrogel for Miniature pH Sensors. Adv. Mater..

[27] Lin X., Su J., Lin H., Zhou S.-F., Sun X., Liu B., Zeng M. (2019). Carbon nanoparticles with oligonucleotide probes for a label-free sensitive antibiotic residues detection based on competitive analysis. Sci. Rep. UK.

[28] Peppas N.A., Merrill E.W. (1977). Crosslinked poly(vinyl alcohol) hydrogels as swollen elastic networks. J. Appl. Polym. Sci..

[29] Deak A., Csapo E., Juhasz A., Dekany I., Janovak L. (2018). Anti-ulcerant kynurenic acid molecules intercalated Mg/Al-layered double hydroxide and its release study. Appl. Clay Sci..

[30] Jimenez-Vergara A.C., Lewis J., Hahn M.S., Munoz-Pinto D.J. (2018). An improved correlation to predict molecular weight between crosslinks based on equilibrium degree of swelling of hydrogel networks. J. Biomed. Mater. Res. B.

